# Porcine jejunal-derived extracellular vesicles participate in the regulation of lipid metabolism

**DOI:** 10.1186/s40104-025-01185-x

**Published:** 2025-04-07

**Authors:** Yaotian Fan, Haibin Deng, Jiahao Zhu, Junyi Luo, Ting Chen, Jiajie Sun, Yongliang Zhang, Qianyun Xi

**Affiliations:** Guangdong Provincial Key Laboratory of Animal Nutrition Control, National Engineering Research Center for Breeding Swine Industry, College of Animal Science, South China Agricultural University, No. 483 Wushan Road, Guangzhou, 510642 China

**Keywords:** Intestinal extracellular vesicles, Landrace piglets, Lantang piglets, Lipid metabolism, MiR-30b-5p, Porcine primary adipocytes

## Abstract

**Background:**

Regulating the regional deposition of fat is crucial for improving the carcass characteristics of pigs. The intestine, as an important organ for lipid absorption and homeostasis maintenance, secretes various biological signals that participate in the crosstalk between the intestine and adipose tissue. Extracellular vesicles, as novel extracellular genetic factors that mediate metabolic signal exchange among multiple tissues, have emerged as a hotspot and breakthrough in revealing the mechanisms of physiological homeostasis. However, how extracellular vesicles regulate the intestinal-adipose signaling axis, especially in relation lipid metabolism and deposition is still unclear. Thus, in the current study, intestinal extracellular vesicles from Chinese fat-type piglets of Lantang and typical lean-type piglets of Landrace were isolated and identified, and to reveal the regulatory mechanisms of lipid metabolism via intestinal extracellular vesicles in mediating intestinal-adipose crosstalk.

**Results:**

We isolated and identified intestinal extracellular vesicles from the jejunum of 3-day-old Lantang and Landrace piglets (LT-EVs and LD-EVs) and further investigated their effects on lipid accumulation in porcine primary adipocytes. Compared to LD-EVs, LT-EVs promoted lipid deposition in porcine primary adipocytes, with intestinal-derived miRNAs playing a critical role in the crosstalk between the intestine and adipose tissue. Further analysis of extracellular vesicles-derived miRNA sequencing revealed that miR-30b-5p, enriched in LD-EVs, is involved in the regulation of lipid metabolism. Notably, the enrichment of miR-30b-5p in extracellular vesicles derived from IPEC-J2 cells also influenced lipid metabolism. Mechanistically, the targeted binding of miR-30b-5p and *FMO3* may be critical for the extracellular vesicle-mediated regulation of lipid metabolism.

**Conclusions:**

Our findings suggest that jejunal-derived extracellular vesicles play a critical role in regulating lipid metabolism, and the regulatory effect of extracellular vesicles from obese piglets was higher than that of lean piglets. Furthermore, the different expression of miRNAs, such as miR-30b-5p, in intestinal extracellular vesicles may be the key to determining lipid deposition phenotypes across the two pig breeds.

**Graphical abstract:**

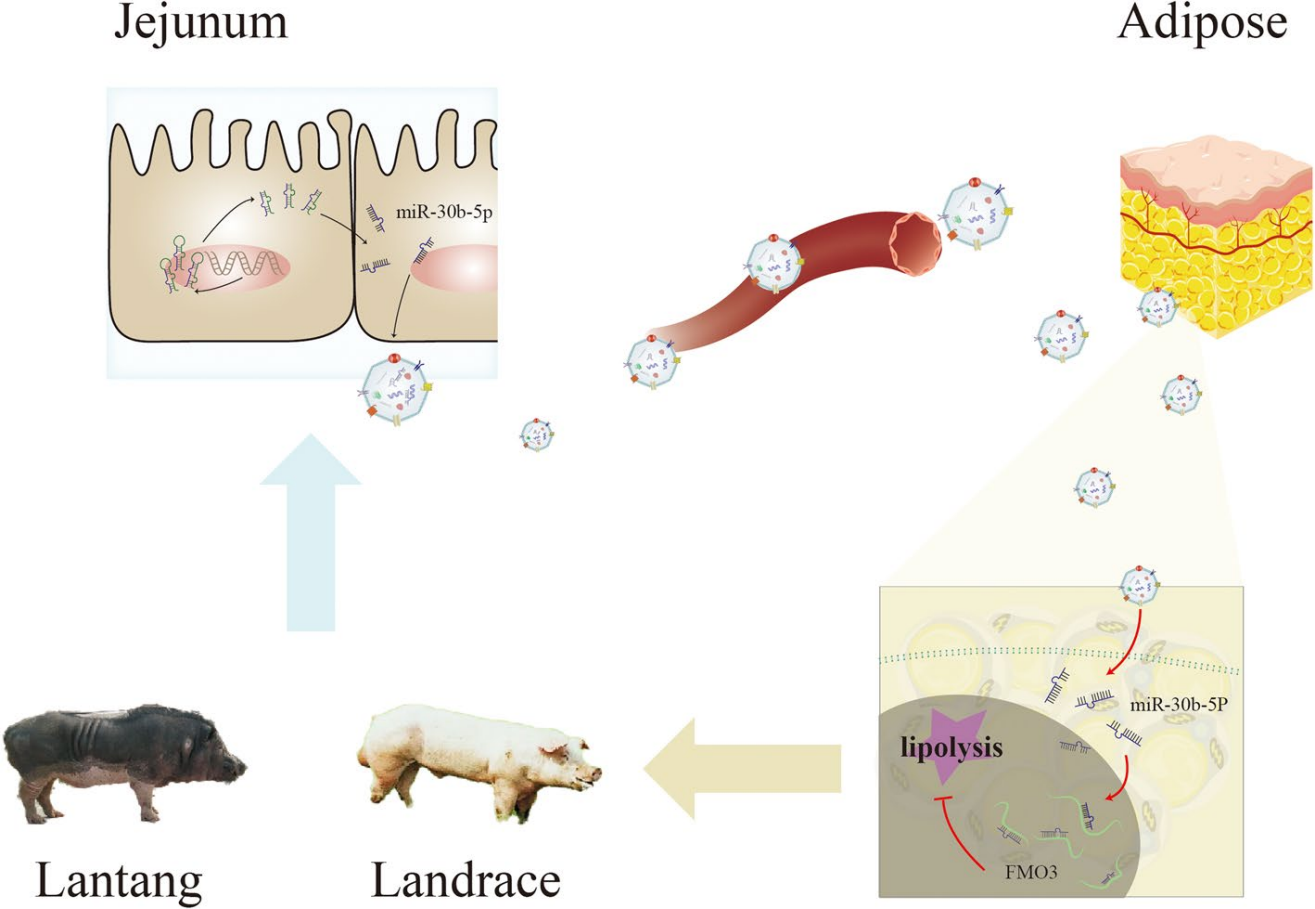

## Introduction

Pork is the most widely consumed source of protein for humans and is a globally popular type of meat [1]. However, the promotion of intensive production models and the development of genetic breeding technologies pose challenges to pork quality [2]. Among the various indicators used to evaluate pork quality, intramuscular fat content is an important factor influencing both the quality and flavor of pork [3]. On the one hand, the content of intramuscular fat is closely related to the characteristics of the meat [4]. On the other hand, the quantity and composition of fatty acids in muscle play crucial roles in meat quality, determining its nutritional value and flavor [5]. Therefore, regulating fat metabolism and its regional deposition is essential for enhancing the quality of pork carcasses.

The intestine, as an important organ for transmitting nutritional signals from external sources, plays a significant role in metabolic regulation [6]. The function of the intestine is critical in regulating the intestine-adipose signaling axis [7]. The intestine is a major site for lipid absorption, participating in the uptake of both dietary lipids and endogenous lipids derived from sloughed intestinal epithelial cells and bile produced by the liver [8]. Moreover, various biological signals generated from the breakdown of nutrients in the intestine (such as hormones, signaling peptides, and cytokines) are involved in the metabolic regulation between the intestine and adipose tissue. Thus, exploring new mechanisms by which intestinal signals participate in lipid metabolism regulation is important for controlling lipid metabolism patterns in pigs and enhancing pork carcass quality.

Emerging evidence indicates that extracellular vesicles (EVs), as novel signaling factors mediating inter-organ signal regulation, play a role in lipid metabolism [9–13]. Functional signals derived from EVs, such as ncRNAs, mRNAs, proteins, and lipids, have been confirmed to participate in the metabolic cross-talk regulation of multiple tissues as novel extracellular genetic signaling molecules. Thus, exploring the crosstalk between the intestine and adipose tissue through signaling derived from intestinal EVs to uncover the mechanisms behind pork quality trait formation and energy homeostasis regulation via new signaling pathways that regulate the intestine-adipose axis has significant research implications.

To explore the functional role of miRNAs derived from intestinal EVs in mediating lipid deposition regulation, this study isolated intestinal EVs from pigs with different fat deposition types (Lantang and Landrace piglets) and performed bioinformatics prediction and functional validation of the miRNA sequences. The results indicate that the loading of miR-30b-5p in the intestinal EVs of Landrace piglets can activate lipid degradation regulation by modulating the expression of FMO3. These findings reveal the differences in lipid metabolism between pigs with different types of fat deposition from the perspective of intestinal EVs, providing new insights for improving the quality of pork carcasses and achieving regional fat deposition.

## Materials and methods

### Animals and samples

Eight newborn Chinese fat-type piglets of Lantang (LT) and eight typical lean-type piglets of Landrace (LD) selected for this study were purchased from a breeding farm (Xinfeng County, Shaoguan City, Guangdong, China). On the third day after the birth of the piglets, blood samples were collected from the jugular vein of four healthy suckling LT male piglets (0.74 ± 0.18 kg) and four healthy suckling LD male piglets (1.38 ± 0.23 kg). The collected blood was centrifuged at 3,000 r/min for 15 min at 4 °C. The supernatant was subsequently stored at −20 °C for the analysis of triglyceride (TG), total cholesterol (T-CHO), and low-density lipoprotein cholesterol (LDL-C) contents, following the instructions of commercial products (Nanjing Jiancheng Bioengineering Institute, China). After the piglets were euthanized, jejunal tissue and adipose tissue from each piglet were collected. The adipose tissue and a portion of the jejunal tissue were rapidly frozen in liquid nitrogen for further analysis. Moreover, the remaining jejunal tissues were stored in PBS for the isolation of jejunal EVs. The jejunal contents were collected from four healthy weaning LT male piglets (4.58 ± 0.29 kg) and four healthy weaning LD male piglets (7.11 ± 0.15 kg) at 28 d. After the euthanasia, the jejunal contents from each piglet were harvested separately and snap-frozen in liquid nitrogen for further analysis. The experiment was approved by the Animal Care Institution and Ethics Committee of South China Agriculture University. All animal experiments were conducted at South China Agriculture University and adhered to the animal experiment policy of South China Agriculture University, China (SYXK2014-0136).

### Primary porcine preadipocyte isolation and culture

The isolation and culture of primary porcine preadipocytes followed previously reported procedures [14, 15]. Landrace piglets were anesthetized and euthanized by intracardiac exsanguination, followed by disinfection of the entire body with alcohol. Under sterile conditions, the subcutaneous fat was then exposed, and the adipose tissue was separated. The isolated adipose tissue was placed in precooled DMEM/F12 culture medium (Gibco, USA) containing 1% penicillin–streptomycin (Sigma, USA). The separated adipose tissue was rinsed three times in DMEM/F12 culture medium and subsequently placed in standard DMEM/F12 culture medium. The adipose tissue was chopped into pieces (approximately 1 mm^3^) and transferred to a 50-mL centrifuge tube (Guangzhou Jet Bio-Filtration Co., Ltd., China), where it was centrifuged at 800 × *g* for 5 min at 4 °C to remove the culture medium. The tissues were treated with a digestion solution containing 0.1% type-I collagenase (1 mg/mL, Gibco, USA) for 90 min at 37 °C. After digestion, the undigested tissue was filtered through a 200-mesh filter and then centrifuged at 800 × *g* for 5 min to remove the supernatant. The cell pellet was resuspended in DMEM/F12 medium. The suspension was centrifuged again at 800 × *g* for 5 min, and the supernatant was discarded. Ten milliliters of erythrocyte lysis buffer was added to the pellet, which was then resuspended and left at room temperature for 10 min [15]. After filtering through a 400-mesh filter, the sample was centrifuged at 800 × *g* for 5 min, and the rinsing process was repeated twice. Finally, the cells were resuspended in DMEM/F12 culture medium containing 10% FBS (Gibco, USA) and 1% penicillin–streptomycin and seeded at a density of 1 × 10^5^ cells/mL. After 24 h of culture, the medium was changed and replaced every 2 d. Once the cells reached confluence, lipid induction was performed on the primary preadipocytes using DMEM/F12 culture medium containing 10% FBS, 1% penicillin–streptomycin, 50 nmol/L insulin (MCE, USA), 50 nmol/L dexamethasone (Sigma, USA), 50 μmol/L oleic acid (Sigma, USA), and 0.5 mmol/L octanoic acid (Sigma, USA). The intestinal EVs of Lantang and Landrace piglets (at a protein concentration of 10 μg/mL) or methimazole (10 μmol/L, Sigma, USA) were added starting on the first day of induction and then every other day for a total of 8 d. On the final day after induction, the cells were collected for the assessment of related indicators. All the preadipocytes used in the study were isolated from a single Landrace piglet.

### Isolation and characterization of intestinal EVs

The isolation of intestinal EVs was based on previous reports [16]. Briefly, the jejunal tissue was washed in precooled PBS to remove contents, followed by rinsing the inverted intestine with cold PBS (5 min, three times). The intestinal segments were cut into 2–3 cm pieces and collected in a 50-mL centrifuge tube. A solution of DTT dissolved in PBS (Dithiothreitol, 10 mmol/L, Sangon, Shanghai, China) was then added, and the mixture was placed on ice, reacting at 100 r/min on a discoloration shaker for 5 min. After removing the DTT solution, the cleaned intestinal tissue was placed in 8 mmol/L EDTA solution (EDTA dissolved in PBS, Sangon, Shanghai, China) and reacted at 60 r/min on a shaker for 30 min at 4 °C. Following the reaction, the solution was shaken vigorously for 5 min and then centrifuged at 3,000 r/min for 15 min at 4 °C, collecting the supernatant, which contained the crude intestinal EVs. For further separation of the intestinal EVs, the crude intestinal EVs were centrifuged at 12,000 × *g* for 30 min at 4 °C and filtered through a 0.22-µm filter. The filtrate was then placed in an ultracentrifuge tube and centrifuged at 120,000 × *g* for 2 h at 4 °C, yielding a pellet of the prepared jejunal EVs. Finally, the pellet of intestinal EVs was dissolved in sterile PBS buffer, and the dissolved jejunal EVs were filtered through a 0.22-µm filter and aliquoted into centrifuge tubes for subsequent indicator detection and experimental validation.

### Transmission electron microscopy analysis of EVs

The intestinal EVs were placed on ice and diluted to a ratio of 1:10 for subsequent analysis. After thorough mixing, 10 μL of the intestinal EVs were placed on a copper grid coated with poly (methyl methacrylate), and the excess EV solution at the edges of the copper grid was removed. The grid was incubated at room temperature for 5 min, after which uranyl acetate was added dropwise to the grid for negative staining for 1 min. The prepared copper grid was then placed under a transmission electron microscope (Talos L120C, FEI Company, USA) to observe the morphology of the isolated intestinal EVs.

### Nanoparticle tracking analysis of EVs

The intestinal EVs were diluted to a ratio of 1:50 for nanoparticle tracking analysis. A NanoSight LM10 particle size analyzer (Malvern, the UK) was used to determine the concentration and analyze the particle size of the intestinal EVs. A constant rate injection pump was used to inject 1 mL of intestinal EVs into the instrument. Tracking was performed at room temperature for 1 min, with manual adjustments made to the parameters and values read to maintain consistency within the sample. Each sample was measured three times, and the NanoSight analysis tool was used to analyze the captured particle number and concentration.

### TG determination

The TG concentration of primary porcine preadipocytes was measured after 8 days of adipocyte differentiation. Tissue TG assay kit (BC0625, Solarbio, China) was purchased to determine the TG content according to the manufacturer’s instructions.

### Oil Red O and Nile red staining

Oil Red O staining was performed as previously described [10]. Briefly, induced differentiated primary porcine preadipocytes were washed three times with PBS. Then, the cells were fixed with 4% paraformaldehyde (Sangon, Shanghai, China) for 30 min. Subsequently, the cells were treated with Oil Red O staining solution [diluted 2:3 (v/v) with PBS and filtered through a 0.22-μm membrane] (Sigma, USA) for 30 min. After treatment, the cells were washed three times with PBS and observed under a microscope for lipid droplets.

The Nile red staining was performed as previously described [17]. The cells were washed three times with PBS and then incubated with Nile red solution (200 nmol/L) (Sigma, USA) at room temperature for 10 min. After treatment, the cells were washed three times with PBS and observed under a fluorescence microscope (Nikon Ti-U738270) for lipid droplets.

### PKH67 labeled EVs uptake

Freshly isolated intestinal EVs from Lantang and Landrace piglets were labeled with PKH67 according to the manufacturer’s protocol (BB-441112, BestBio, China). The PKH67 fluorescent probe was diluted to a ratio of 1:250. Then, 100 μL of intestinal EVs was mixed with the PKH67 solution and incubated in the dark for 30 min at room temperature. The labeled EVs were centrifuged at 120,000 × *g* for 2 h, and the particles were suspended with PBS. The PKH67 labeled EVs were added to the cell culture medium and incubated for 12 h. DAPI was used as a nuclear marker, and the cells were observed under a fluorescence microscope.

### MiRNA-seq analysis of EVs

TruSeq Small RNA Sample Prep Kits (Illumina, San Diego, USA) were used for the isolation and construction of small RNA sequencing libraries from porcine intestinal EVs with different types of fat deposition. The constructed library was sequenced using Illumina Hiseq 2000/2500 (LC-Bio Technology, Hangzhou, China) with a single ended read length of 1 × 50 bp. miRNA data analysis was performed using the ACGT101 miR software (LC Sciences, Houston, Texas, USA). Then, the software of TargetScan (v5.0) and MiRanda (v3.3a) was performed to predict target genes for significantly different miRNAs of intestinal EVs of Lantang and Landrace piglets, separately. Screen the target genes predicted by the two software according to the scoring criteria of each software. In the TargetScan algorithm, target genes with a context score percentage less than 50 are removed, whereas in the MiRanda algorithm, target genes with maximum free energy greater than −10 are removed (TargetScan_stcore > 50 and Miranda-Energy < −10). Finally, the intersection of these two software was taken as the final target gene for differentially expressed miRNAs, and functional annotation analyses such as GO and KEGG were performed.

### Transfection of porcine primary preadipocytes and IPEC-J2 cells

RNAiPro Transfection Reagent was used to transfect the miR-30b-5p mimic (S: 5′-UGUAAACAUCCUACACUCAGCU-3′; AS: 5′-CUGAGUGUAGGAUGUUUACAUU-3′), the miR-30d mimic (S: 5′-UGUAAACAUCCCCGACUGGAAGCU-3′; AS: 5′-CUUCCAGUCGGGGAUGUUUACAUU-3′), and negative control (NC) (S: 5′-UUCUCCGAACGUGUCACGUTT-3′; AS: 5′-ACGUGACACGUUCGGAGAATT-3′) into porcine primary preadipocytes and IPEC-J2 cells. Briefly, porcine primary preadipocytes and IPEC-J2 cells were seeded in 6-well plates and transfected with 100 nmol/L miR-30b-5p mimic, miR-30d mimic or NC per well according to commercial product instructions (MK4018, MIKX, Shenzhen, China). After 48 h post-transfection, the differentiation of porcine primary preadipocytes continued. After 24 h post-transfection, IPEC-J2 cells were washed with PBS for three times, and relevant indicators were measured.

### Dual luciferase assay

Luciferase vectors (pmirGLO) containing wild-type or mutant-type *FMO3* untranslated region were amplified and cloned (TSINGKE, Beijing, China). Then, luciferase vectors were co-transfected with miR-30b-5p mimic or NC into 293 T cells using Lipofectamine 2000 (Thermo Fisher). According to the manufacturer’s instructions, the luciferase activities were determined by a Dual Luciferase Reporter Assay System (Promega). The sequences are as follows:

*FMO3*-WT: 5′-GCTAGCGGGGCTGCCATCCCCACAGCTGACCTGCAGGCCCGCTGGGTGGTTAAAGTGTTTACAAGTAAGTGGGTTATTATGTCTTTCATTCATTTAGTCAACAAATGTCGAC-3′.

*FMO3*-MUT: 5′-GCTAGCGGGGCTGCCATCCCCACAGCTGACCTGCAGGCCCGCTGGGTGGTTAAAGACAAATGAAGTAAGTGGGTTATTATGTCTTTCATTCATTTAGTCAACAAATGTCGAC-3′.

### 16S rRNA sequence analysis

A fecal genomic DNA extraction kit was used to extract the total DNA from jejunal contents to analyze the microbial community. A 1% agarose gel and a micro-nucleic acid concentration measuring device were subsequently used to assess the concentration and purity of the extracted DNA. The 16S V4 region of bacteria was PCR amplified using primers 515F and 806R. The PCR products were purified using a general DNA purification kit and then used for library construction. The constructed library was quantified using Qubit and qPCR, and once deemed acceptable, sequenced on a NovaSeq 6000 (Novogene, Beijing, China) using PE 250 sequencing. The raw reads were filtered with fqstp software to obtain high-quality clean tags. Taxonomic annotation was performed based on the reference genome (SILVA_138.1) using the Mothur algorithm.

### Western blot analysis

The total protein from porcine primary adipocytes was incubated with RIPA lysis buffer for 30 min at 4 °C. Then, the lysate was centrifuged at 12,000 × *g* for 10 min to remove the precipitate. The total protein concentration was determined using the BCA Protein Assay kit (23225, Thermo Fisher, USA), and 20 μg of total protein was separated by reducing SDS-PAGE on 10% Bis–Tris gels, transferred to 0.45-μm PVDF membranes and probed with different primary antibodies: FASN (1:1,500, R381582, Zen-Bioscience, Chengdu, China), ACC (1:1,000, A19627, ABclonal, Wuhan, China), CD36 (1:1,000, 18836-1-AP, Proteintech, Wuhan, China), PPARγ (1:1,000, 340844, Zen-Bioscience), C/EBPα (1:1,000, R30014, Zen-Bioscience), FATP4 (1:1,000, HA720001, HUABIO, Hangzhou, China), FABP4 (1:1,000, ET1703-98, HUABIO), DGAT1 (1:1,000, ER1907-35, HUABIO), Alix (1:1,500, A2215, ABclonal, Wuhan, China), TSG101 (1:1,500, A2215, ABclonal), CD63 (1:1,000, 25682-1-AP, Proteintech), A33 (1:1,500, A3608, ABclonal), FMO3 (1:1,500, A19222, ABclonal), β-Tubulin (1:5,000, AP0064, Bioworld, Nanjing, China), and β-actin (1:5,000, AP0060, Bioworld). The membranes were incubated with primary antibodies overnight at 4 °C. Then, the protein bands were incubated with goat anti-rabbit IgG secondary antibody (1:50,000, 14708, CST, USA) for 1 h at room temperature and visualized using the Tanon 5200 system. The grayscale value was determined by ImageJ software analysis.

### Statistical analysis

Graphs were generated using GraphPad Prism 9.0 software. All data were statistically analyzed using SPSS 22.0 software (IBM, Chicago, IL, USA). The significance of differences between the groups was assessed using one-way analysis of variance (ANOVA) and Student’s *t*-tests. All the data are presented as mean ± standard error of the mean (SEM), *P* < 0.05 indicated significant differences.

## Results

### Comparison of lipid metabolism differences between Lantang and Landrace piglets

**Fig. 1:**
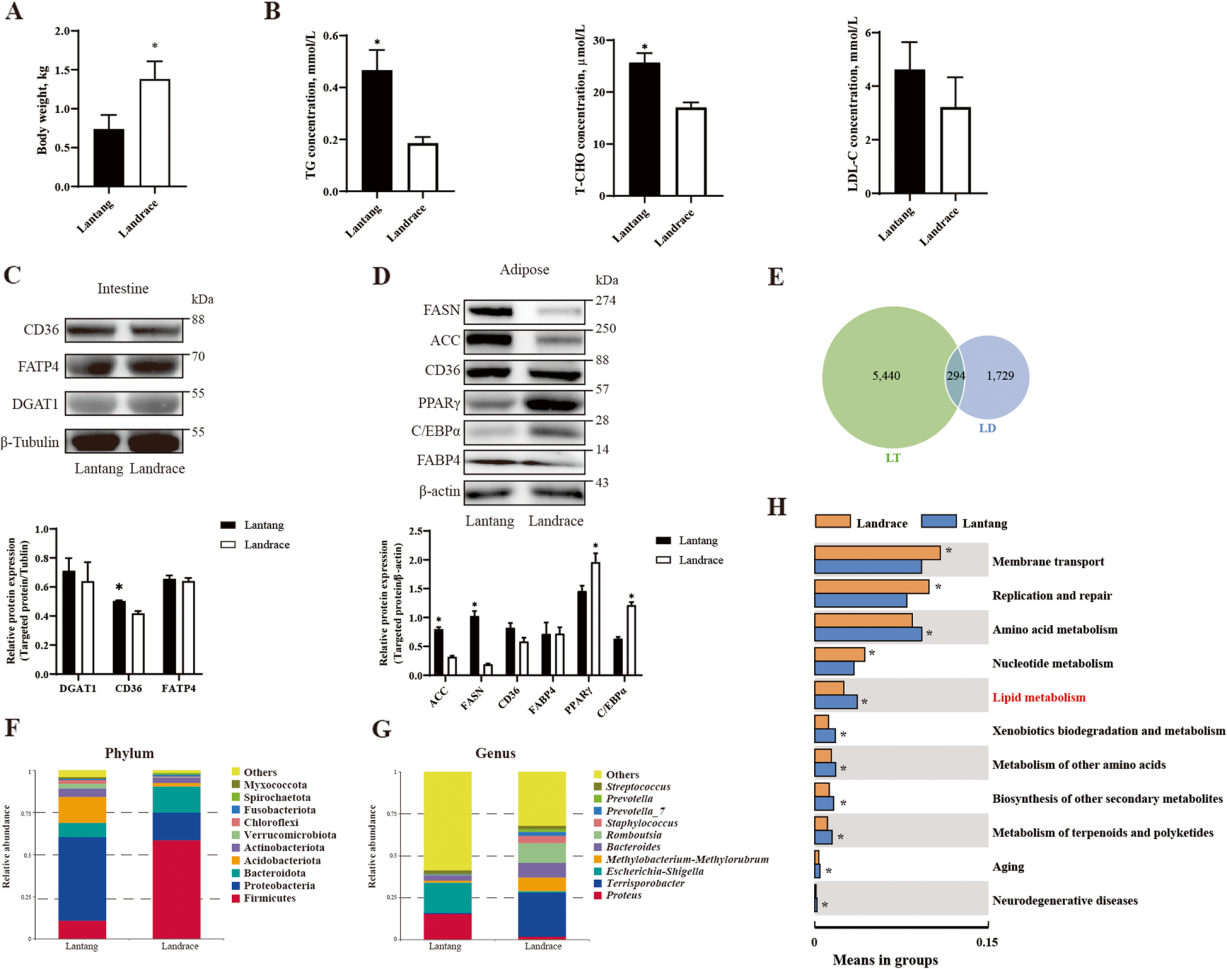
The characterization of lipid metabolism in Lantang and Landrace piglets (*n* = 4). **A** The body weight of 3-day-old Lantang and Landrace piglets. **B** The TG, T-CHO, and LDL-C contents in serum of 3-day-old Lantang and Landrace piglets. **C** The expression of lipid transport related proteins in the jejunum of 3-day-old Lantang and Landrace piglets. **D** The expression of lipid metabolism related proteins in the adipose of 3-day-old Lantang and Landrace piglets. **E** Venn diagram of bacterial OTUs in jejunal microbiota of 28-day-old Lantang and Landrace piglets. **F** Comparison of phylum level proportional abundance of jejunal microbiota between 28-day-old Lantang and Landrace piglets. **G** Comparison of genus level proportional abundance of jejunal microbiota between 28-day-old Lantang and Landrace piglets. **H** The Tax4Fun function prediction of different jejunal microbiota in 28-day-old Lantang and Landrace piglets. Values are presented as means ± SEM. * represent significant difference (*P* < 0.05). Abbreviations: TG, triglyceride; T-CHO, total cholesterol; LDL-C, low-density lipoprotein cholesterol

In the current study, we compared the differences in body weight, intestinal lipid absorption, serum biochemistry, adipose tissue metabolism, and the gut microbiota between Lantang and Landrace piglets. The results indicated that the body weight of Landrace piglets at 3 d after birth was significantly higher than that of Lantang piglets (*P* < 0.05) (Fig. 1A). Furthermore, the determination of lipid metabolism-related enzyme activity in serum revealed that the serum levels of TG and TCHO in Lantang piglets were significantly higher than those in Landrace piglets (*P* < 0.05), while there was no significant difference in serum LDL-C levels between Lantang and Landrace piglets (*P* > 0.05) (Fig. 1B). Moreover, the protein expression of CD36 in the jejunal tissue of Lantang was significantly higher than that in Landrace piglets (*P* < 0.05), whereas the expression levels of DGAT1 and FATP4 in the jejunal tissues had no significant differences (*P* > 0.05) (Fig. 1C). To further investigate the differences in lipid metabolism between Lantang and Landrace piglets, we collected subcutaneous fat from the piglets and conducted relevant measurements. The study found that the protein expression of FASN and ACC in the jejunal tissue of Lantang piglets were significantly higher than those in Landrace piglets (*P* < 0.05), while the protein expression of PPARγ and C/EBPα was significantly increased in Landrace piglets (*P* < 0.05). There was no significant difference in the expression of CD36 and FABP4 in the adipose tissues of Lantang and Landrace piglets (*P* > 0.05) (Fig. 1D). The crosstalk between the intestine and adipose is multiple and complex. Gut microbiota plays an important role in regulating lipid metabolism. To understand whether gut microbiota in Lantang and Landrace piglets involved in the regulation of lipid metabolism regulation, the 16S rRNA sequencing was performed. Considering the vulnerability of 3-day-old piglet jejunal microbiota to colonization and microbial homeostasis, which may affect the judgment of the results, our study selected the jejunal contents of 28-day-old piglets to determine the correlation between gut microbiota and lipid metabolism in Lantang and Landrace piglets. The 16S rRNA sequencing results from the jejunal microbes of Lantang and Landrace piglets at 28 d indicated that there were 294 common OTUs in the jejunal contents of both pig breeds, with 5,440 unique OTUs expressed in the jejunal contents of Lantang piglets and 1,729 unique OTUs expressed at higher levels in the jejunal contents of Landrace piglets (Fig. 1E). At the phylum level, Firmicutes, Proteobacteria, and Bacteroidota were the dominant phylum in the jejunal contents of piglets. The stacked histogram showed that, compared to the microbial distribution in the jejunal contents of Lantang piglets, the proportion of Firmicutes was significantly increased, while the proportion of Proteobacteria was significantly decreased in the jejunal contents of Landrace piglets (*P* < 0.05) (Fig. 1F). At the genus level, *Proteus*, *Terrisporobacter*, and *Escherichia-Shigella* were the dominant genera, with a significantly increased proportion of *Terrisporobacter* and a significantly decreased proportion of *Proteus* and *Escherichia-Shigella* in the jejunal contents of Landrace piglets (*P* < 0.05) (Fig. 1G). To explore the functional changes in metabolic regulation associated with the differential microbial communities, we conducted the Tax4Fun functional prediction targeting these differential microbial communities. The results indicated that the major functions of the differential microbes in the jejunal contents of Lantang and Landrace piglets focused on 11 pathways: membrane transport, replication and repair, amino acid metabolism, nucleotide metabolism, lipid metabolism, biodegradation and metabolism of exogenous organic matter, other amino acid metabolism, secondary metabolite biosynthesis, terpenoid compound and polyketide metabolism, aging, and neurodegenerative diseases (*P* < 0.05) (Fig. 1H). The regulatory pathway changes associated with these differential microbial communities indicate significant differences in the metabolic patterns of pigs with different fat deposition types, suggesting that these differing bacteria play a key role in maintaining the metabolic functional homeostasis.

### Isolation and identification of intestinal EVs in Lantang and Landrace piglets

**Fig. 2:**
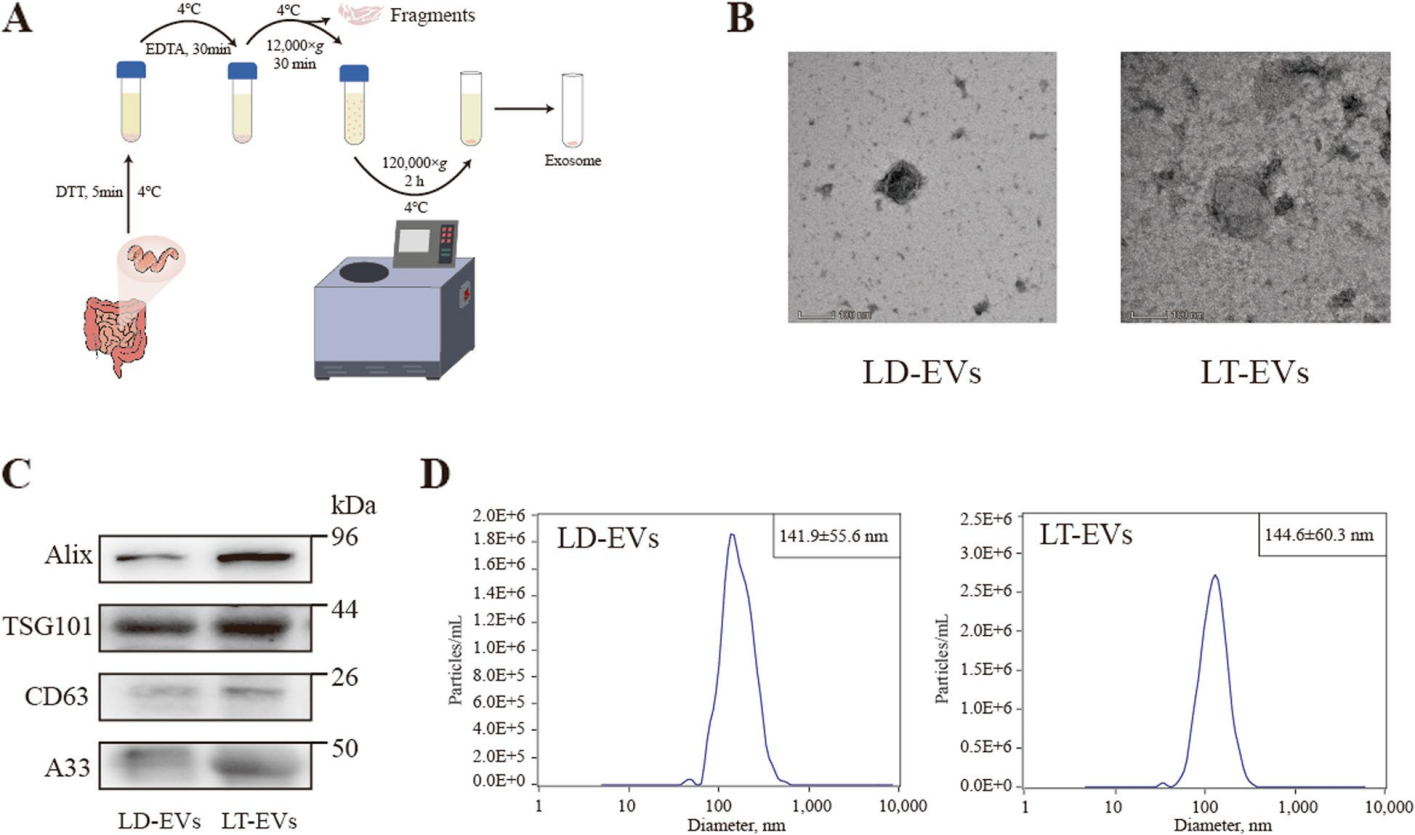
Isolation and identification of porcine intestinal extracellular vesicles (*n* = 4). **A** A flow chart of intestinal extracellular vesicles from Lantang and Landrace piglets. **B** Observation of intestinal extracellular vesicles in Lantang and Landrace piglets by transmission electron microscopy. **C** Western blot detection of extracellular vesicles membrane proteins. **D** Nanoparticle tracking analysis of intestinal extracellular vesicles in Lantang and Landrace piglets. Abbreviations: LT-EVs, intestinal extracellular vesicles form Lantang; LD-EVs, intestinal extracellular vesicles form Landrace. Each assay was repeated in triplicate

To explore whether the differences in lipid metabolism between Lantang and Landrace piglets are related to jejunal EVs, our study first extracted jejunal EVs from 3-day-old Lantang and Landrace piglets (Fig. 2A), and identified the EVs derived from the jejunal tissues of both piglet breeds. Electron microscopy of the intestinal EVs from both Langtang and Landrace piglets revealed that the isolated EVs possessed a vesicular structure (Fig. 2B). Additionally, Western blot analysis of EV-derived marker proteins (Alix, CD63, TSG101) and intestinal epithelial cell marker protein (GPA33) indicated that the jejunal EVs collected from both Lantang and Landrace piglets by ultracentrifugation were characteristic proteins of intestinal EVs (Fig. 2C). The particle size analysis of EVs revealed that the size of EVs derived from Lantang piglets (LT-EVs) was predominantly approximately 144.6 nm, while the size of EVs derived from Landrace (LD-EVs) was mainly approximately 141.9 nm, indicating no significant difference in the average particle size of jejunal EVs between the two piglet breeds (Fig. 2D). In summary, this study successfully isolated jejunal EVs from Lantang and Landrace piglets for the first time and identified them for subsequent functional research.

### Lipid metabolism regulation by LT-EVs and LD-EVs

**Fig. 3:**
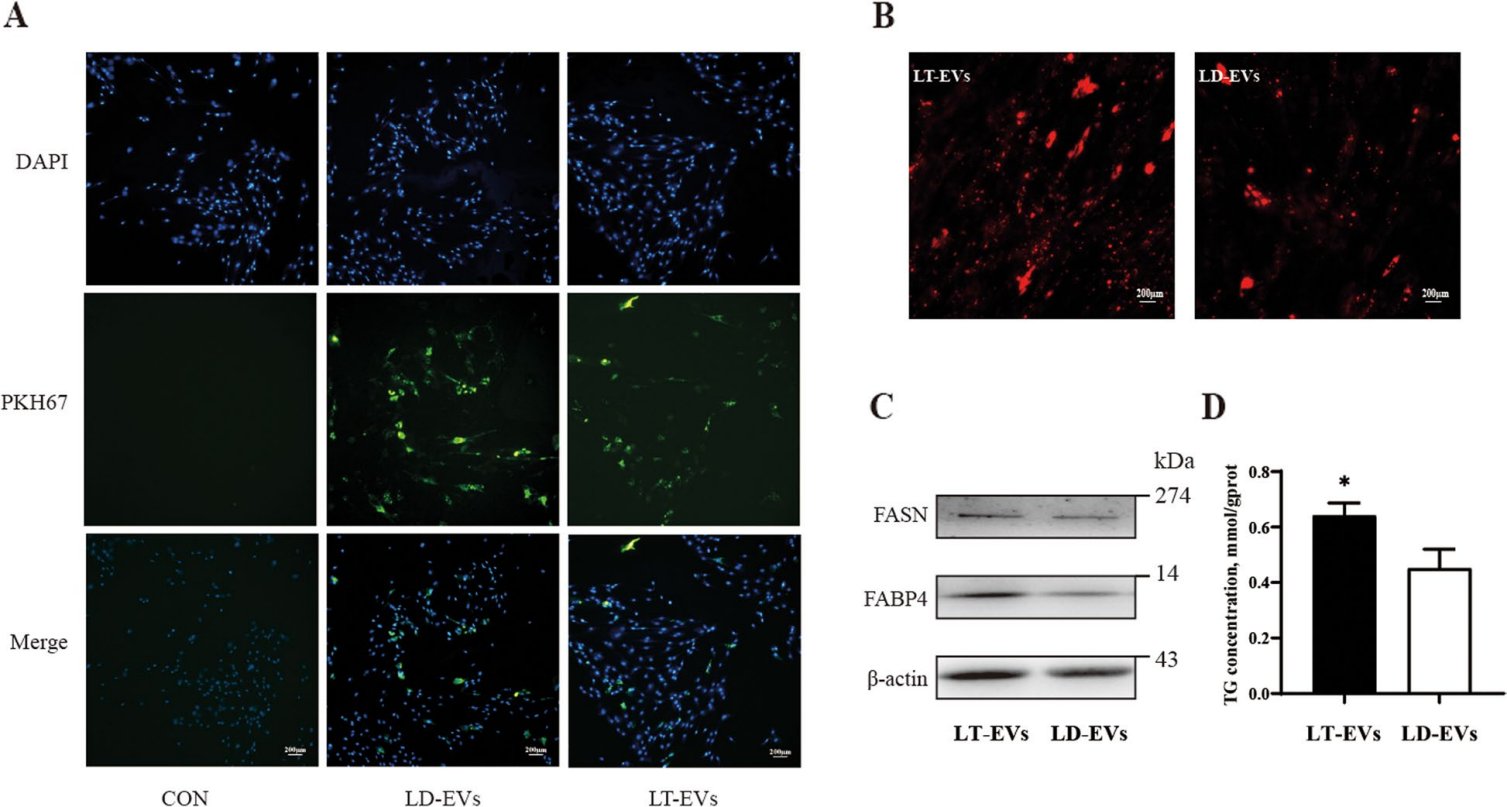
The uptake and lipid regulation function of intestinal extracellular vesicles in Lantang and Landrace piglets (*n* = 3). **A** Characterization of PKH67 (green) labeled intestinal extracellular vesicles from Lantang and Landrace piglets in porcine primary adipocytes. **B** Oil Red O staining of lipid droplets in primary porcine adipocytes stimulated by intestinal extracellular vesicles of Lantang and Landrace piglets. **C** Western blotting detection of the key lipogenic proteins. **D** The TG content of primary porcine adipocytes stimulated by intestinal extracellular vesicles of Lantang and Landrace piglets. Values are presented as means ± SEM. * represent significant difference (*P* < 0.05). Abbreviations: LT-EVs, intestinal extracellular vesicles form Lantang; LD-EVs, intestinal extracellular vesicles form Landrace; TG, triglyceride. Each assay was repeated in triplicate

To investigate the uptake capacity of porcine primary preadipocytes for pig intestinal EVs, this study added PKH67-labeled jejunal EVs from Lantang and Landrace piglets to a culture system of porcine primary preadipocytes and observed them after 12 h of treatment. The results showed that after 12 h of treatment with intestinal EVs, the presence of labeled intestinal EVs from Lantang and Landrace piglets could be effectively observed in porcine primary preadipocytes (green, Fig. 3A), and there was no difference in the uptake capacity of jejunal EVs between Lantang and Landrace piglets by porcine primary preadipocytes. Next, we verified the functional effects of intestinal EVs on porcine primary preadipocytes. The results indicated that LT-EVs could induce a rapid increase in the number of lipid droplets in porcine primary preadipocytes compared to LD-EVs (Fig. 3B). Additionally, LT-EVs enhanced the expression of lipid differentiation-related proteins (FASN and FABP4) in porcine primary adipocytes (Fig. 3C). Moreover, the measurement of TG content in adipocytes also showed that, compared to LD-EVs, LT-EVs were more beneficial for increasing the TG content in the primary adipocyte culture system (*P* < 0.05) (Fig. 3D). These results confirm that intestinal EVs are involved in the regulation of lipid metabolism and that there are differences in lipid metabolism regulation between intestinal EVs from different pig breeds.

### miRNA-seq analysis of LT-EVs and LD-EVs

**Fig. 4:**
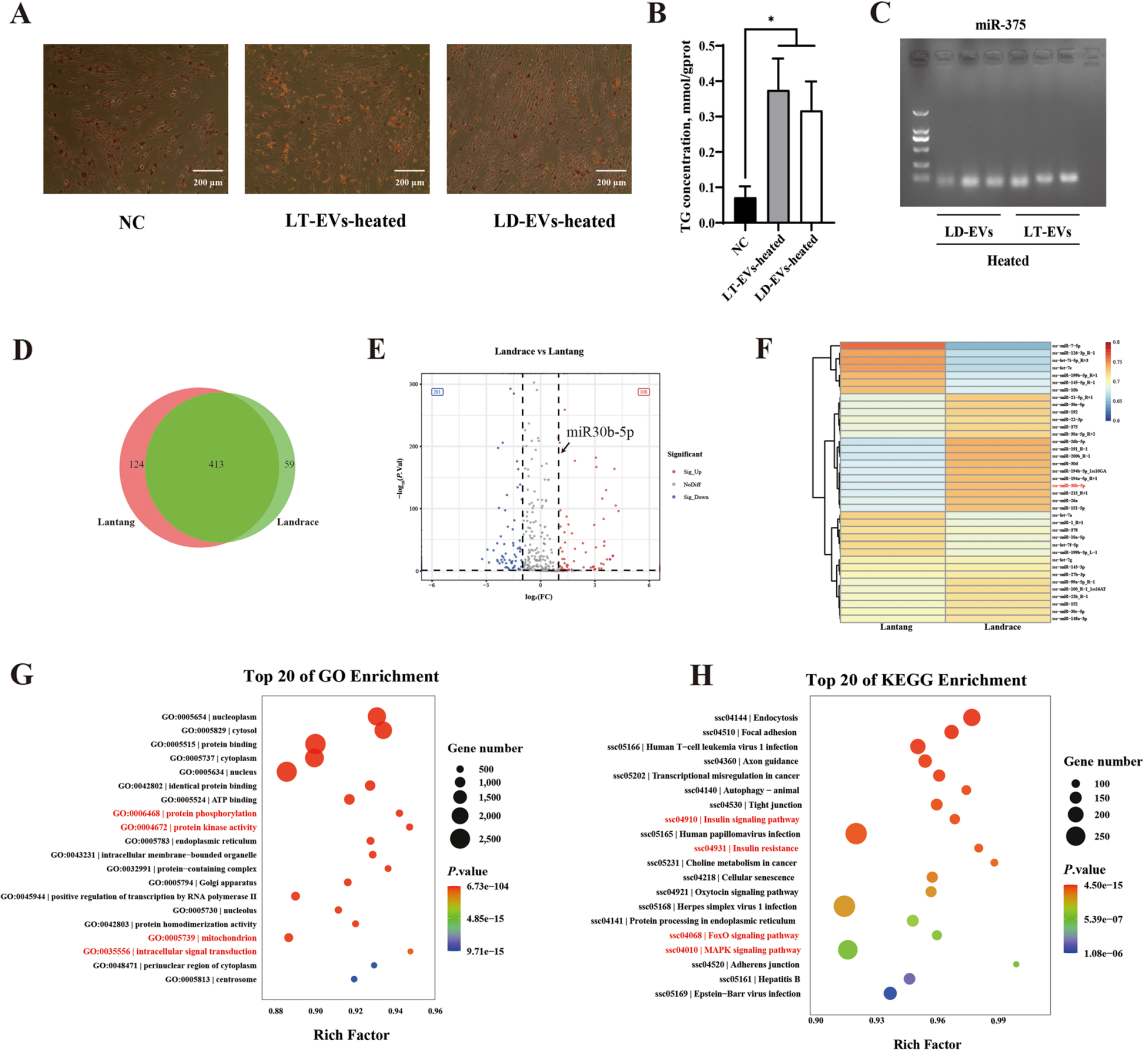
The differentially expressed miRNAs analysis between porcine intestinal extracellular vesicles of Lantang and Landrace. **A** Oil Red O staining of lipid droplets in primary porcine adipocytes stimulated by heat treated intestinal extracellular vesicles of Lantang and Landrace piglets (*n* = 3). **B** The TG content of primary porcine adipocytes stimulated by heat treated intestinal extracellular vesicles of Lantang and Landrace piglets (*n* = 3). **C** The observation of miR-375 gel electrophoresis in heat treated intestinal extracellular vesicles of Lantang and Landrace piglets (*n* = 3). **D** Venn diagram of differentially expressed miRNAs in intestinal extracellular vesicles of Lantang and Landrace piglets. **E** Volcanic diagram of differentially expressed miRNAs in intestinal extracellular vesicles of Lantang and Landrace piglets. **F** Heat map of differentially expressed miRNAs in intestinal extracellular vesicles of Lantang and Landrace piglets. **G** GO enrichment analysis of differentially expressed miRNAs in intestinal extracellular vesicles of Lantang and Landrace piglets. **H** KEGG enrichment analysis of differentially expressed miRNAs in intestinal extracellular vesicles of Lantang and Landrace piglets. Values are presented as means ± SEM. * represent significant difference (*P* < 0.05). Abbreviations: LT-EVs, intestinal extracellular vesicles form Lantang; LD-EVs, intestinal extracellular vesicles form Landrace; TG, triglyceride. Each assay was repeated in triplicate

To further investigate the role of component differences in jejunal EVs from Lantang and Landrace piglets in regulating porcine primary preadipocytes, we first subjected intestinal EVs to heat treatment (80 °C for 60 min) to inactivate complex functional protein molecules within the EVs. Interestingly, heat-inactivated jejunal EVs from both Lantang and Landrace piglets still promoted an increase in the number of lipid droplets in porcine primary adipocytes (Fig. 4A). Additionally, heat-inactivated EVs significantly elevated the TG content in porcine primary adipocytes (*P* < 0.05), but there was no difference in the differentiation function of LT-EVs and LD-EVs after heat inactivation (*P* > 0.05) (Fig. 4B). These results suggest that the presence of heat-resistant components in EVs may mediate lipid metabolism regulatory functions. Since miRNAs are known to have heat-resistant properties, this study examined the expression of the characteristic miR-375 (specifically encapsulated in host EVs, not in microbial EVs) in LT-EVs and LD-EVs following heat treatment through gel electrophoresis [7]. The results showed that miR-375 remained expressed in LT-EVs and LD-EVs after heat inactivation, indicating that EVs-derived miRNAs can effectively resist environmental changes. The miRNAs from the heat-treated LT-EVs and LD-EVs may be involved in the regulation of lipid metabolic signals (Fig. 4C). To further investigate the role of miRNAs in LT-EVs and LD-EVs in mediating lipid metabolism regulation, we performed sequencing analysis of the jejunal EVs-derived miRNAs. The results indicated that a total of 413 miRNAs were co-expressed in LT-EVs and LD-EVs, with 124 unique miRNAs expressed in LT-EVs and 59 specific miRNAs expressed in LD-EVs (Fig. 4D). A total of 309 miRNAs were found to have differential expression (fold change > 2 or fold change < 0.5, *P* < 0.05) between the two breeds, of which 201 were highly expressed in LT-EVs, while 108 were more highly expressed in LD-EVs (Fig. 4E). Subsequently, we conducted a heatmap analysis on the 37 differentially expressed miRNAs that exhibited high expression in the jejunal EVs of Lantang and Landrace piglets (Fig. 4F). The top 10 highly expressed miRNAs in LD-EVs were: miR-215, miR-200b, miR-26b-5p, miR-194b-5p, miR-194a-5p, miR-191, miR-30d, miR-30b-5p, miR-26a, and miR-192; while the top 10 highly expressed miRNAs in LT-EVs were: miR-7-5p, let-7i-5p, miR-126-3p, let-7e, miR-199b-5p, miR-145-5p, miR-10b, let-7a, miR-1, and let-7f-5p. The results confirmed significant differences in the loading of miRNAs in jejunal EVs from Lantang and Landrace piglets. To further understand the functions of the differentially expressed miRNAs in jejunal EVs from Lantang and Landrace piglets, we performed GO enrichment analysis and KEGG pathway analysis on these miRNAs. The results indicated that the GO enrichment analysis identified several pathways of involvement for miRNAs in LT-EVs and LD-EVs, including protein phosphorylation, protein kinase activity, mitochondrion, and intracellular signal transduction (Fig. 4G). Furthermore, the KEGG pathway functional enrichment analysis of the different miRNAs indicated that the differential miRNAs in EVs are primarily involved in the regulation of metabolic pathways related to the insulin signaling pathway, the insulin resistance, the FoxO signaling pathway, and the MAPK signaling pathway (Fig. 4H). Overall, these results demonstrate that miRNAs from LT-EVs and LD-EVs play a role in regulating the metabolic functions of adipose tissue.

### Intestinal EVs-derived miR-30b-5p participates in lipid metabolism regulation

**Fig. 5:**
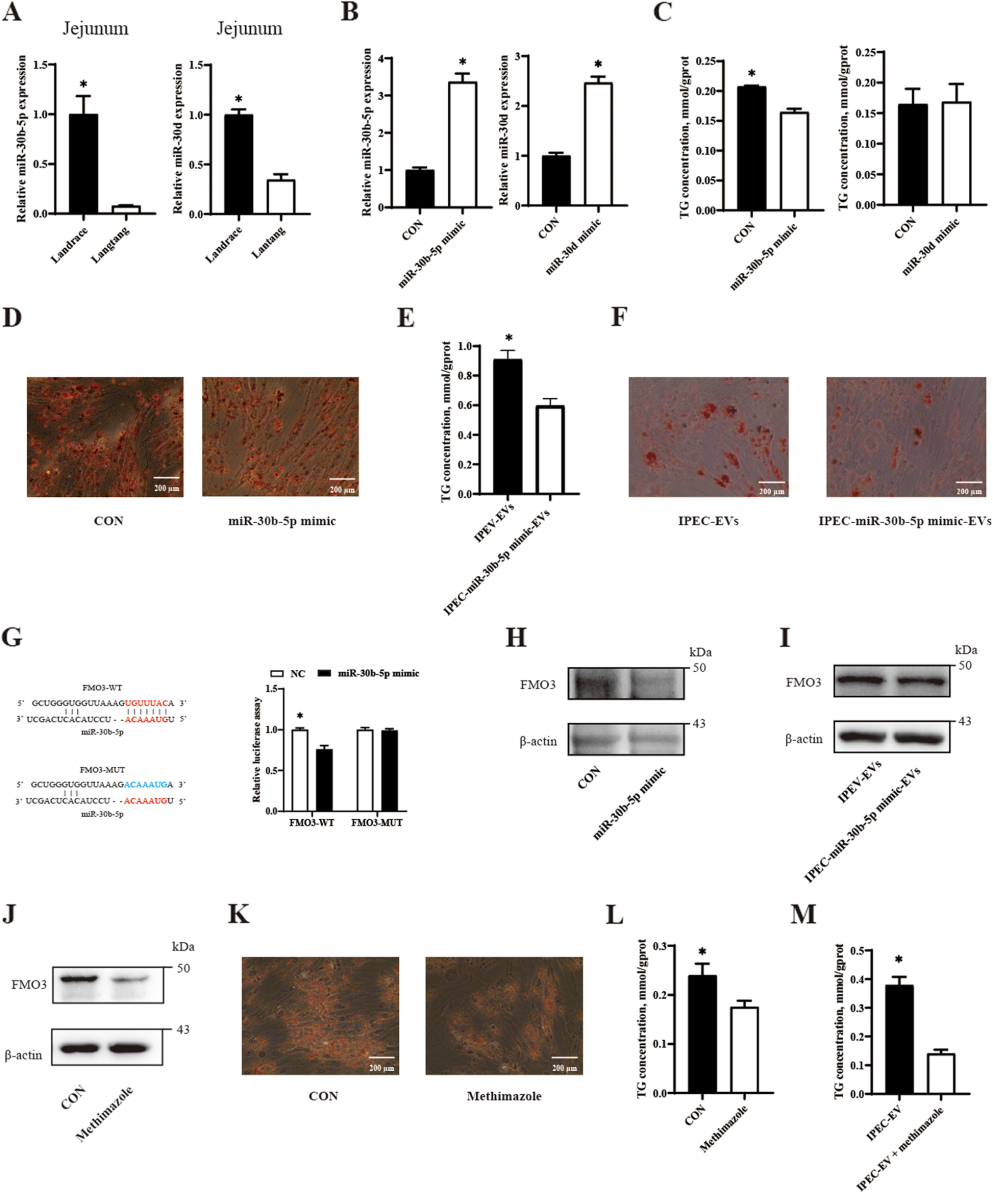
Intestinal extracellular vesicles derived miR-30b-5p participate in lipid metabolism regulation (*n* = 3). **A** The relative expression of miR-30b-5p and miR-30d in the jejunum of Lantang and Landrace piglets. **B** The transfection efficiency of miR-30b-5p mimic or miR-30d mimic in porcine primary adipocytes. **C** The TG content of primary porcine adipocytes transfected by miR-30b-5p mimic or miR-30d mimic. **D** Oil Red O staining of lipid droplets in primary porcine adipocytes transfected by miR-30b-5p mimic. **E** The TG content of primary porcine adipocytes stimulated by miR-30b-5p mimic transfected IPEC-J2 derived extracellular vesicles. **F** Oil Red O staining of lipid droplets in primary porcine adipocytes stimulated by miR-30b-5p mimic transfected IPEC-J2 derived extracellular vesicles. **G** The dual luciferase assay showed miR-30b-5p targeted *FMO3*. **H** The FMO3 protein expression of primary porcine adipocytes stimulated by miR-30b-5p mimic. **I** The FMO3 protein expression of primary porcine adipocytes stimulated by miR-30b-5p mimic transfected IPEC-J2 derived extracellular vesicles. **J** The FMO3 protein expression of primary porcine adipocytes stimulated by methimazole. **K** Oil Red O staining of lipid droplets in primary porcine adipocytes stimulated by methimazole. **L** The TG content of primary porcine adipocytes stimulated by methimazole. **M** The TG content of primary porcine adipocytes co-stimulated by methimazole and IPEC-J2 derived extracellular vesicles. Values are presented as means ± SEM. * represent significant difference (*P* < 0.05). Abbreviations: EVs, intestinal extracellular vesicles; TG, triglyceride. Each assay was repeated in triplicate

The miR-30 family is recognized as key miRNAs regulating lipid metabolism. In our current study, we found that miR-30b-5p and miR-30d are differentially expressed in LT-EVs and LD-EVs. To further investigate whether and how the miR-30 family participate in lipid metabolism regulation of porcine primary preadipocytes, we first measured the expression of miR-30b-5p and miR-30d in the intestinal tissues of Lantang and Landrace piglets. The results showed that, consistent with the changes in EVs, the expression level of miR-30b-5p and miR-30d in the jejunal tissues of Landrace piglets was significantly higher than that in Lantang piglets (*P* < 0.05) (Fig. 5A). These results suggest that the differential expression of miR-30b-5p and miR-30d in the jejunum may account for the variations in miR-30b-5p and miR-30d expression between LT-EVs and LD-EVs. To validate the regulatory function of miR-30 family in lipid metabolism, we transfected porcine primary preadipocytes with miR-30b-5p or miR-30d mimics, and then measured the transfection efficiency of the miR-30b-5p or miR-30d mimic in porcine primary adipocytes. The addition of the miR-30b-5p and miR-30d mimics significantly increased the expression levels of miR-30b-5p and miR-30d in porcine primary preadipocytes (*P* < 0.05) (Fig. 5B). The lipid metabolic functions of porcine primary adipocytes transfected with the miR-30b-5p and miR-30d mimics were subsequently investigated. The results indicated that transfection with the miR-30b-5p mimics significantly reduced the TG content (*P* < 0.05) (Fig. 5C) and the number of lipid droplets (Fig. 5D) in porcine primary adipocytes, while transfection with the miR-30d mimics had no effect on the TG content in porcine primary adipocytes (*P* > 0.05) (Fig. 5C). To further confirm the role of miR-30b-5p loaded in intestinal EVs in lipid metabolism regulation, IPEC-J2 cells were transfected with the miR-30b-5p mimics, and EVs were harvested. The results showed that EVs derived from IPEC-J2 cells overexpressing miR-30b-5p significantly decreased the TG content in porcine primary adipocytes (*P* < 0.05) (Fig. 5E). Additionally, Oil Red O staining in porcine primary adipocytes also indicated that the EVs from IPEC-J2 cells overexpressing miR-30b-5p reduced the number of lipid droplets (Fig. 5F). To further explore how miR-30b-5p in EVs regulated lipid metabolism, this study used TargetScan and Miranda software to predict the target genes of miR-30b-5p. The results indicated that the seed region of miR-30b-5p could target the *FMO3* gene. Then, the dual luciferase assay demonstrated that miR-30b-5p targeted *FMO3* (Fig. 5G). Furthermore, the transfection of the miR-30b-5p mimics into porcine primary preadipocytes also reduced the expression level of FMO3 in porcine primary adipocytes (Fig. 5H). Additionally, the loading of miR-30b-5p in intestinal-derived EVs effectively reduced the expression of FMO3 in porcine primary adipocytes (Fig. 5I). To investigate the correlation between FMO3 and lipid metabolism in porcine primary adipocytes, methimazole (a specific inhibitor of FMO3) was used to stimulate porcine primary adipocytes. We first investigated the inhibitory efficiency of methimazole on FMO3. The results indicated that methimazole reduced the expression of FMO3 in porcine primary adipocytes (Fig. 5J). The Oil Red O staining in porcine primary adipocytes also indicated that methimazole reduced the number of lipid droplets (Fig. 5K). Moreover, methimazole significantly decreased the TG content in porcine primary adipocytes (*P* < 0.05) (Fig. 5L). To further investigate whether the regulation of lipid deposition by EVs was associated with FMO3, we co-stimulated porcine primary adipocytes with methimazole and IPEC-J2 derived EVs. The results demonstrated that co-stimulation with methimazole and IPEC-J2 derived EVs significantly reduced TG content compared to stimulation with IPEC-J2 derived EVs alone in porcine primary adipocytes (*P* < 0.05) (Fig. 5M), indicating that FMO3 regulated lipid deposition mediated by intestinal EVs. In summary, miR-30b-5p loaded in porcine intestinal EVs could participate in regulating lipid metabolism by affecting the expression of the *FMO3* gene in porcine primary adipocytes.

## Discussion

Regulating fat metabolism and deposition is crucial for improving the carcass quality of pigs. The intestine, as an essential site for lipid absorption, plays a critical role in maintaining homeostasis for lipid metabolism [18]. The differences in endogenous and exogenous signals caused by genetic and environmental exposure pose significant challenges to intestinal homeostasis. The relationship between the heterogeneity of exogenous signals and lipid metabolism has been reported [19, 20]. In the current study, the changes in the intestinal microbial composition of Lantang and Landrace piglets were consistent with previous reports, indicating that intestinal microbes participate in reshaping metabolic characteristics [21, 22]. Notably, endogenous crosstalk regulation plays a dominant role in the remodeling of metabolic characteristics [23]. Chinese local breeds and European pigs represent two independently evolved branches, displaying distinct metabolic characteristics [24]. Previous studies have shown that the differential expression of the *CIDE* gene in pigs with varying fat deposition types determines the development of lipid deposition [25]. Moreover, the expression heterogeneity of the IGF system in Lantang and Landrace piglets also contributes to the regulation of growth performance and carcass quality [26]. Our previous study revealed that the differences in lipid metabolism regulation between Lantang and Landrace piglets are highly correlated with gene expression heterogeneity [22]. In the current study, we found that the gene heterogeneity of 3-day-old Lantang and Landrace piglets is involved in the regulation of lipid metabolism fate. Therefore, the regulation of crosstalk between intestinal and fat signals, which is based on genetic signaling, may be crucial for influencing lipid metabolism in pigs.

As mediators of signaling regulation between various tissues, intestinal EVs serve as novel extracellular genetic regulatory factors involved in lipid metabolism [9]. Notably, the effective uptake of EVs by target tissues or cells is pivotal for their functional role. Recent studies have shown that intestinal epithelium-derived EVs can directly influence adipocytes via the bloodstream [9]. This finding is consistent with the observed effective uptake of EVs by adipocytes in the current study. Moreover, some studies indicate that changes in lipid distribution within intestinal EVs can affect lipid metabolism, as they participate in regulating intestinal homeostasis through TGF-β signaling [27]. Similar to the metabolic functional characterization of intestinal EVs induced by a high-fat diet [9], the intestinal EVs from Lantang piglets demonstrated greater differentiation capability than those from Landrace piglets. The metabolic regulatory function of EVs is based on the heterogeneous regulation of their cargo. Thus, the heterogeneous expression of cargo in the intestinal EVs of both Lantang and Landrace has attracted attention.

In fact, the miRNAs carried by intestinal EVs are widely involved in glucose and lipid metabolism processes [28]. Intestinal EVs-derived miRNAs may serve as significant signaling molecules that mediate the intestinal-fat axis [29]. In the current study, porcine intestinal EVs treated at 80 °C for 1 h still presented lipid regulatory functions, suggesting that heat-resistant miRNAs may mediate lipid metabolism regulation. Sequencing of intestinal EVs-derived miRNAs revealed that miR-30b-5p is loaded in the intestinal EVs of Landrace piglets. Furthermore, the expression of miR-30b-5p in porcine primary adipocytes and intestinal derived EVs, mediates the lipid metabolism regulation. Notably, the miR-30 family has been reported to broadly participate in lipid metabolism regulation [30, 31]. Increased expression of miR-30e enhances the β-oxidation of fatty acids, whereas suppression of the miR-30 family reduces fatty acid β-oxidation [32]. Furthermore, miR-30b-5p can participate in lipid metabolism regulation by modulating PPARγ expression [33]. Thus, miR-30b-5p in EVs may be a key signaling molecule in the differential regulation of lipid metabolism mediated by porcine intestinal EVs.

Many studies indicate that the binding of miRNAs to their target genes is fundamental for their metabolic regulatory functions. In the current study, we focused on the bioinformatics prediction of differential miRNAs identified in LT-EVs and LD-EVs. These results indicate that the highly expressed miR-30b-5p in the intestinal EVs of Landrace piglets can target and bind to FMO3. Then, the dual luciferase assay further confirmed the target relationship between *FMO3* and miR-30b-5p. FMO3 is a central integrative factor in the metabolism of cholesterol and triglycerides in the liver, as well as in inflammation and endoplasmic reticulum stress. Members of the FMO family can act as regulators of endogenous energy homeostasis involved in controlling lipid metabolism [34, 35]. The expression of the *FMO3* gene is closely correlated with the execution of lipid metabolic functions in the liver. Emerging evidence has reported that the absence of the *FMO3* gene leads to a reduced capacity for lipid accumulation in the liver of ducks [36]. Additionally, mutations in the *FMO3* gene in laying hens have been shown to decrease the risk of lipid metabolic diseases [37]. Recent research indicated that the expression of FMO3 in adipose tissue also affects the phenotype of fat deposition [38]. In the current study, we found that the expression of FMO3 in porcine primary adipocytes participates in the regulation of lipid deposition and FMO3 regulates lipid deposition mediated by intestinal EVs. Thus, the enrichment of miR-30b-5p in porcine intestinal EVs is the key mediator of intestinal and fat signal crosstalk.

## Conclusions

In summary, this study enriches the understanding of the role of miR-30b-5p in the regulation of lipid metabolism and reveals that miRNA-30b-5p loaded in the intestinal EVs of Landrace piglets can participate in the lipid breakdown process by regulating the FMO3 signaling pathway in porcine adipocytes. These findings provide new insights into the differential regulatory functions of lipid metabolism mediated by the intestinal EVs of piglets with different fat deposition types.

## Data Availability

16S rRNA sequencing data have been deposited in the NCBI BioProject database under accession numbers PRJNA1168669. MicroRNA sequencing data have been deposited in the NCBI BioProject database under accession numbers SRR30883132.

## Abbreviations

EVs: Extracellular vesicles
LD: Landrace
LD-EVs: Intestinal extracellular vesicles form Landrace
LDL-C: Low-density lipoprotein cholesterol
LT: Langtang
LT-EVs: Intestinal extracellular vesicles form Lantang
T-CHO: Total cholesterol
TG: Triglyceride
